# Complete Genome Sequence of the Multiresistant Taxonomic Outlier *Pseudomonas aeruginosa* PA7

**DOI:** 10.1371/journal.pone.0008842

**Published:** 2010-01-22

**Authors:** Paul H. Roy, Sasha G. Tetu, André Larouche, Liam Elbourne, Simon Tremblay, Qinghu Ren, Robert Dodson, Derek Harkins, Ryan Shay, Kisha Watkins, Yasmin Mahamoud, Ian T. Paulsen

**Affiliations:** 1 Département de biochimie et de microbiologie, and Centre de recherche en infectiologie, Université Laval, Québec, Québec, Canada; 2 Department of Chemistry and Biomolecular Sciences, Macquarie University, Sydney, New South Wales, Australia; 3 J. Craig Venter Institute, Rockville, Maryland, United States of America; University of Hyderabad, India

## Abstract

*Pseudomonas aeruginosa* PA7 is a non-respiratory human isolate from Argentina that is multiresistant to antibiotics. We first sequenced *gyrA*, *gyrB*, *parC*, *parE*, *ampC*, *ampR*, and several housekeeping genes and found that PA7 is a taxonomic outlier. We report here the complete sequence of the 6,588,339 bp genome, which has only about 95% overall identity to other strains. PA7 has multiple novel genomic islands and a total of 51 occupied regions of genomic plasticity. These islands include antibiotic resistance genes, parts of transposons, prophages, and a pKLC102-related island. Several PA7 genes not present in PAO1 or PA14 are putative orthologues of other *Pseudomonas* spp. and *Ralstonia* spp. genes. PA7 appears to be closely related to the known taxonomic outlier DSM1128 (ATCC9027). PA7 lacks several virulence factors, notably the entire TTSS region corresponding to PA1690-PA1725 of PAO1. It has neither *exoS* nor *exoU* and lacks *toxA*, *exoT*, and *exoY*. PA7 is serotype O12 and pyoverdin type II. Preliminary proteomic studies indicate numerous differences with PAO1, some of which are probably a consequence of a frameshift mutation in the *mvfR* quorum sensing regulatory gene.

## Introduction

*Pseudomonas aeruginosa* is an environmental bacterium that is an opportunistic pathogen of humans. It causes wound and burn infections as well as respiratory infections, the latter especially in cystic fibrosis (CF) patients. *P. aeruginosa* is known for its antibiotic resistance, notably its efflux systems but also antibiotic modifying enzymes, and for its multiple virulence factors, enabling formation of biofilms and infection of multiple host species. The first completely sequenced strain of *P. aeruginosa* was the laboratory strain PAO1 [1]. Its sequence revealed a genetic complexity, including a large number of secretion and efflux systems, that is consistent with its ability to thrive in a wide variety of environments. The sequence of the more virulent strain PA14 [2] revealed that additional genes in the latter, related to survival in diverse environmental conditions, clustered into genomic islands, while the “core genome” (about 90% of total genes) was highly similar to that of PAO1. It was shown that the increased virulence of PA14 was both multifactorial and combinatorial, and that virulence for eukaryotic hosts has its determinants in the core genome. These observations were extended with the addition of the genomes of two CF isolates, PA2192 and C3719 [3], showing a conserved core genome and sets of genomic islands, the latter coding for auxiliary genes (metabolic, virulence, and resistance genes, and prophages) for survival in particular host environments. The sequence of the Liverpool Epidemic Strain LESB58 [4] showed that both the core genome and genomic islands (including prophages) are involved in *in vivo* competitiveness.

*P. aeruginosa* PA7 is a non-respiratory clinical isolate from Argentina. It is one of 10 non-respiratory isolates that were collected for their unusual resistance patterns by Microcide Pharmaceuticals Inc. (Mountain View, CA). Preliminary sequence data from some resistance and housekeeping genes showed that PA7 is a taxonomic outlier. The complete genomic sequence of PA7 was determined. As with the other sequenced strains, there is a core genome, whose genes show considerable divergence with those of the other sequenced strains. There are a total of 51 occupied genomic islands, including 18 novel ones. While many antibiotic resistance genes are present, several virulence factor genes are lacking, notably for the type III secretion system that enables the injection of toxins into host cells.

## Results

### Extended Resistance Spectrum of *P. aeruginosa* PA7

PA7 is highly resistant to third generation cephalosporins (Minimal Inhibitory Concentration [MIC] for ceftazidime of 128 µg/ml), monobactams (MIC for aztreonam of 64 µg/ml), and fluoroquinolones (FQ) (MIC for ciprofloxacin of 128 µg/ml) (Table 1). The strain is also resistant to piperacillin, carbenicillin, levofloxacin and chloramphenicol, but sensitive to carbapenems (MIC for imipenem of 2 µg/ml and for meropenem of 1 µg/ml). We sequenced the *gyrA*, *gyrB*, *parC*, and *parE* genes in order to determine the source of FQ resistance. We found two mutations typical of FQ resistance, but many more that may or may not be involved in resistance. Other resistance (*ampC*, *ampR*, *mexR* and *oprD*) and housekeeping (*atpD*, *aspS*) genes showed notable divergence with their homologs in PAO1 and PA14. This showed that PA7 is a taxonomic outlier. However, sequences from rRNA genes and the highly conserved *tufB* gene indicated that PA7 is within the species *P. aeruginosa*. To investigate further, we determined the complete genome sequence of PA7 by whole genome shotgun sequencing.

**Table 1:** Minimal inhibitory concentrations (MIC) of some antibiotics for PA7.

Class	Antibiotic	MIC µg/ml
**Penicillins**	piperacillin	>512
	carbenicillin	>512
**Cephalosporins**	ceftazidime	128
	cefoperazone	>32
	cefotaxime	>32
	ceftriaxone	>32
	ceftizoxime	>32
	cefotetan	>32
	cefoxitin	>16
	cefuroxime	>16
	cephalothin	>16
**Monobactam**	aztreonam	64
**Carbapenems**	imipenem	2
	meropenem	1
**Quinolones**	ciprofloxacin	>128
	levofloxacin	>32
	norfloxacin	>8
	gatifloxacin	>8
**Aminoglycoside**	amikacin	>32
**Chloramphenicol**	chloramphenicol	256

### Features of the *P. aeruginosa* PA7 Genome

**Figure 1:**
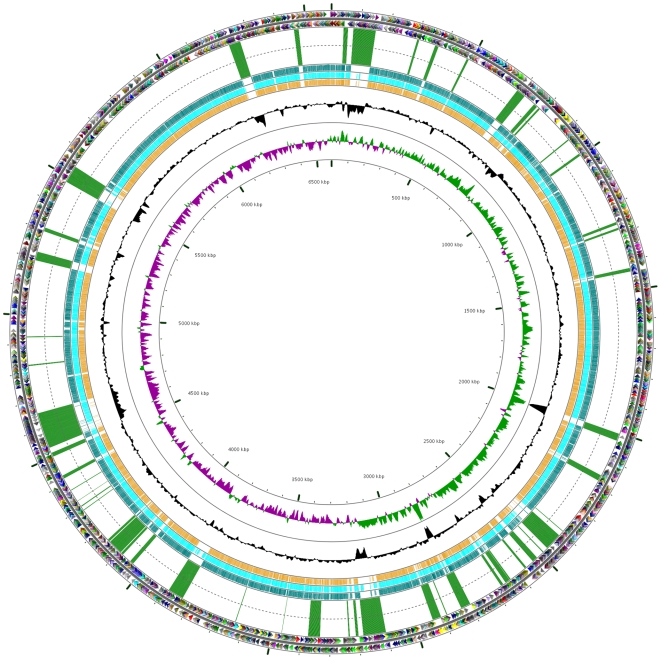
The chromosome of *P. aeruginosa* PA7. The outermost two circles indicate positions of CDSs in plus (circle 1) and minus (circle 2) strands colored by functional category: translation, ribosomal structure, and biogenesis (maroon); transcription (navy); DNA replication, recombination and repair (purple); cell division and chromosome partitioning (brown); posttranslational modification, protein turnover, chaperones (aqua); cell envelope biogenesis, outer membrane (teal); cell motility and secretion (blue); inorganic ion transport and metabolism (orange); signal transduction mechanisms (lavender); energy production and conversion (olive); carbohydrate transport and metabolism (light green); amino acid transport and metabolism (dark green); nucleotide transport and metabolism (fuchsia); coenzyme metabolism (pink); lipid metabolism (red); secondary-metabolite biosynthesis, transport, and catabolism (yellow); general function prediction only (dark grey); function unknown (light grey); and no COG (black). Genomic islands or ‘regions of genomic plasticity’ are indicated by green bars in the third circle; these are in the same order as listed in Table 3 (starting from the 0 kbp mark). Moving toward the center, the following three circles map pairwise blastn alignments (expected threshold = 1e−20) between PA7 and previously sequenced *P. aeruginosa* genomes (circle 4 PAO1 (teal); circle 5 PA14 (aqua); circle 6 LESB58 (orange)). Circle seven shows G+C content (deviation from average), and the eighth circle illustrates G+C skew in green (+) and purple (−). The scale (in kbp) is indicated on the innermost circle. CGview software [43] was used to construct the genome map.

**Figure 2:**
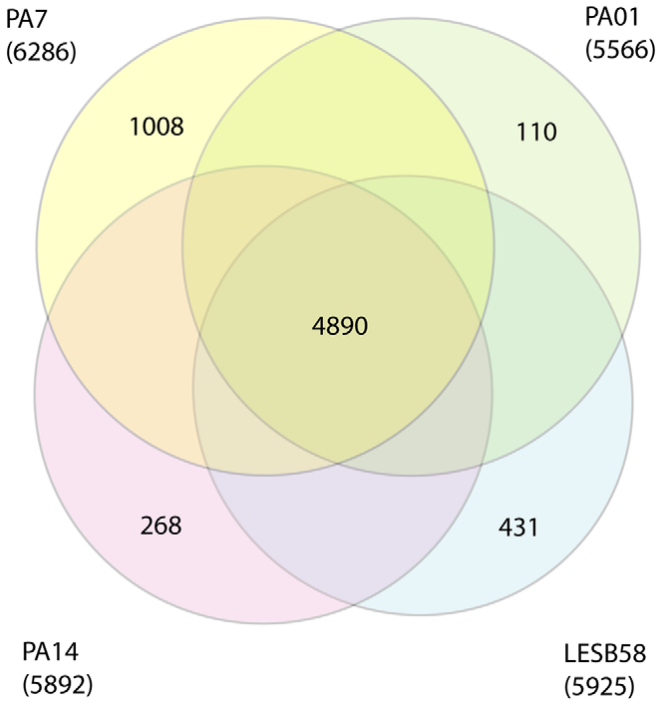
Genomic comparisons of *P. aeruginosa* strains. Venn diagram showing the number of *P. aeruginosa* PA7 predicted proteins with significant similarity (expected threshold = 1e−5) with the predicted products of other *P. aeruginosa* strains PAO1, PA14 and LESB58. The numbers in parentheses represent the total number of predicted proteins for each genome.

The 6,588,339 base pair genome of *P. aeruginosa* PA7 (Figure 1) was predicted to contain 6286 open reading frames (ORFs) representing 90% of total genomic DNA (Figure 2). The average G+C content of the genome was 66.5%, similar to previously sequenced *P. aeruginosa* strains PAO1 [1], PA14 [2] and LESB58 [4] (Table 2). Comparison of PA7 with the other available *P. aeruginosa* genomes [5] in regard to functional category breakdown of coding sequences (CDSs) shows a similar distribution in most groupings with the exception of DNA replication, recombination and repair, which make up a higher proportion of CDSs in this genome (Table 2). This is predominantly due to the large number of additional transposase and integrase genes present in the genomic island regions of PA7. The pairwise percentage identity of syntenous regions between PA7 on the one hand and PAO1, PA14, and LESB58 on the other hand was 93.5%; while that between the latter three strains was 98.7–99.4% (Figure S1, Figure S2 A–G). This is consistent with the sequencing of selected genes that suggested that PA7 is a taxonomic outlier compared with other sequenced *P. aeruginosa* strains.

**Table 2:** General genome features for *P. aeruginosa* strains.

	PA7	PAO1*	PA14*	LESB58*
**Genome Size (bp)**	6,588,339	6,264,404	6, 537, 648	6,601,757
**G+C content**	66.5	66.6	66.3	66.3
**protein coding genes**	6286	5566	5892	5925
**% coding**	89	89	89	88
**structural RNAs** †	75	77	72	81
**pseudogenes**	8	5	none	34
**Assigned function** ‡				
Translation, ribosomal structure and biogenesis	206	205	205	199
Transcription	530	516	537	501
DNA replication, recombination and repair	235	160	185	145
Cell division and chromosome partitioning	37	34	35	34
Posttranslational modification, protein turnover, chaperones	215	200	210	201
Cell envelope biogenesis, outer membrane	260	265	266	261
Cell motility and secretion	152	150	154	149
Inorganic ion transport and metabolism	355	376	377	313
Signal transduction mechanisms	346	337	345	337
Energy production and conversion	336	329	340	330
Carbohydrate transport and metabolism	250	252	249	196
Amino acid transport and metabolism	571	587	590	490
Nucleotide transport and metabolism	105	108	110	104
Coenzyme metabolism	192	191	192	210
Lipid metabolism	245	244	248	234
Secondary metabolites biosynthesis, transport and catabolism	198	205	212	171
General function prediction only	759	756	771	603
Function unknown	503	476	493	500

* The information for *P. aeruginosa* PAO1, PA14 and LESB58 is derived from the NCBI Genome database entry for each strain.

† tRNAs and 16S rRNAs

‡ Based on Cluster of Orthologous Genes (COG) functional categories. An ORF may be assigned multiple functional categories (not all ORFs are assigned a functional category).

### Genomic Homology to Other *P. aeruginosa*

Sequencing of some housekeeping genes of strains in our collection shows that PA7 forms a clade with a small number of strains. Interestingly, these include the strain DSM1128 (ATCC9027) that was excluded from a multilocus sequence typing analysis [6] because of its “unusually high sequence variability in all analyzed loci”, and the strain Pa5196, that has an unusual glycosylation of its type IV pilin, mediated by TpfW and shared by PA7 [7], [8]. PA7's O antigen locus shares a strong similarity to the serotype O12 locus (AF498403) [9], 99.4% over 24 kb. However, its relationship to the European epidemic O12 strains [10] remains unknown.

### Genomic Islands

In addition to the numerous genomic islands in the sequenced *P. aeruginosa* genomes compiled by Mathee et al. (Fig. 2 in [3]), PA7 has 18 novel islands (named RGP63-RGP80) in its genome (Table 3). Four of the genomic islands are putative prophages. RGP66 (30 kb) is similar to the incompletely sequenced phage pspph06 and has parts with similarity to the genomic sequence of *P. syringae pv. phaseolicola* (CP000058) [11]. RGP78 also shows regions of similarity to *P. syringae*. RGP56 shows similarities to sequences from strains PACS458 and PACS10223. RGP60 shows only small regions of similarity to sequences from *P. syringae* and *B. pseudomallei*.

**Table 3:** *P. aeruginosa* PA7 genomic island features.

RGP number*	ORFs included (PSPA7 numbering)	Atypical trinucleotide composition observed?†	inserted near tRNA?	Features of interest encoded in PA7	Other features relevant to mobility‡
RGP46	0043–0046.1	Y	N	hemagglutinins	
**RGP63**	0070–0139	Y	N	mercury resistance cluster, type I restriction modification system	phage integrase at left end, ISPsy6 transposase at right end
RGP1	0285–0291	N	N	ABC transporter	
**RGP64**	0355–0368	N	N		phage integrase at right end
**RGP65**	0473–0476	Y	N		ISxac3 transposase at left end
**RGP66**	0678–0716	N	N	phage-related	phage integrase pseudogene at left end, integrase gene at right end
RGP3	0756–0772	N	N	phage-related	
RGP4	0785.1–0787.1	Y	N		
RGP38	0928–0932	N	N		
RGP44	0988–0995	N	N	two-component system	
**RGP67**	1247–1252.2	N	N		most of these genes translocated relative to PAO1 & PA14
**RGP68**	1268–1272	N	N		region substitutes for exoS region of PAO1
**RGP69**	1407–1420	N	N	type II secretion pathway cluster	
RGP31	1969–1986	Y	N	serotype O12 O-antigen locus	
**RGP70**	2108–2125	N	Y	multidrug efflux system, non-ribosomal protein synthesis genes	
RGP29	2339	N	Y		
RGP56	2363–2436	Y	N	phage-related, DNA adenine methylase, DNA cytosine methylase	phage integrase at left end
RGP43	2460–2464	N	N		
RGP28	2513–2526	Y	Y		
**RGP71**	2550–2555	N	N		
RGP27	2617–2620	N	Y		
**RGP72**	2622–2633	N	Y		
RGP26	2648–2660.2	Y	Y		phage integrase at left end
RGP25	2775–2795	Y	N	hemagglutinins	
RGP24	2834–2837	N	N		
**RGP73**	2858–2861	N	N		
RGP23	3007–3071	Y	N	*cupD* fimbral genes, two component response regulator and sensor kinase	integrases in center
**RGP74**	3089–3094	N	N	iron transport	
RGP22	3114–3118	N	N		
RGP20	3224–3263	N	N		
RGP52	3353	Y	N		
RGP17	3501–3502	N	Y		
**RGP75**	3695–3747	Y	N	conjugal transfer protein cluster, resistance genes, transcriptional regulators	integrase at right end
**RGP76**	3902–3912	N	N		
**RGP77**	3943–3949	Y	N		
RGP15	3953–4007	N	N		includes translocation of region homologous to PA2679-PA2724 to the RGP15 locus
RGP13	4011–4016	N	N	heavy metal efflux system	
RGP12	4148–4149	N	N		
RGP11	4163–4165	N	N		
RGP10	4187	N	N		
RGP47	4228–4230	N	N		
RGP9	4280–4289	N	N		
RGP8	4371–4382	N	Y	ectoine utilisation cluster	
RGP7	4412–4530	Y	Y	type IV B pilus protein cluster, toxin/antitoxin	phage integrase at right end
RGP6	4697–4700	Y	Y		
RGP5	4795–4797	Y	Y		
**RGP78**	5040–5080	Y	N	phage-related	phage integrase at left end, truncated ISPsy11 transposase at right end
RGP60	5143–5161	Y	Y	phage-related	phage integrase at left end
RGP42	5324–5377	Y	Y	truncated integron, streptomycin phosphotransferase, multiple transposon and phage-related genes	integrase at left end
**RGP79**	6033–6063	Y	N	type I restriction-modification system	
**RGP80**	6245–6257	N	N		13-gene island contains 4-gene inversion of PA5456-PA5459

* Bold formatting indicates novel islands (not observed in previously sequenced *P. aeruginosa* strains).

† RGPs where atypical trincleotide composition analysis χ2 values were >500.

‡ left end refers to end of island with the lower bp numbering.

A pKLC102-like island is present in PA7 but is in a different locus (RGP7 rather than RGP41 as in PA14). Its contents are very similar to PAPI-1 except for the presence of five short insertions and the absence of three PAPI-1 specific insertions [12]. PA7 contains six other large islands. RGP63 (75 kb) notably contains genes encoding mercury resistance and a type I restriction-modification system. It shares a 9.8 kb region of identity (4 bp difference) with the PAGI-5 island [13]. RGP56 (59 kb), is partially composed of phage genes as noted above; the rest notably contains a DNA adenine methylase and a DNA cytosine methylase. RGP42 (59 kb), is a “transposon dump” containing elements of Tn*21*, Tn*1721*, and Tn*5393*, as well as 10 genes similar to those of phage Pf1. Notably, there is a truncated integron, with an integrase but no *attI* site, where the *sul1* sulfonamide resistance gene is expressed as a fusion with orf11 [14], as in Tn*610* from *Mycobacterium fortuitum* [15]. RGP23 (33 kb) encodes *cupD* fimbrial genes (see Virulence Factors below). A 12.7 kb region is >99% identical to transposon Tn*4661* (AB375440). RGP75 (53 kb) contains a putative integrated plasmid with several genes encoding conjugative transfer proteins, as well as several resistance genes. These include the three aminoglycoside resistance genes comprising the center of Tn*5*, an isolated integron cassette with the *aacA4* aminoglycoside resistance gene inserted into a secondary site [16], and a chloramphenicol export protein. RGP79 notably encodes a type I restriction-modification system; most of the other genes are of unknown origin.

Other notable genomic islands include: RGP69 (15 kb) encoding a type II secretion pathway; RGP31 (25 kb), the serotype O12 O-antigen locus; RGP70 (20 kb) encoding a multidrug efflux system and non-ribosomal protein synthesis genes, and large segments of which are similar to genomic sequences from *Burkholderia cenocepacia* (CP000959 and CP000459); RGP74, encoding iron transport genes and having 76% overall identity to a region from *Azotobacter vinelandii* DJ [17]; RGP76 (10 kb), 90% identical to a sequence from PACS458 (EU595737); RGP13, encoding heavy metal efflux proteins and similar to sequences from several strains of *Pseudomonas putida*; and RGP8 (14 kb) with ectoine transport genes similar to those of *Pseudomonas stutzeri* and ectoine utilisation genes similar to those of several *Burkholderia spp*.

### Antibiotic Resistance

PA7 is phenotypically resistant to penicillins, cephalosporins, and the monobactam aztreonam, but not to carbapenems (Table 1). It is also resistant to fluoroquinolones, amikacin, and chloramphenicol. Resistance to Beta-lactams is probably due to the *ampC* (class C) and *poxB* (class D) Beta-lactamases encoded by PSPA7_0984 and PSPA7_6316, respectively. The *ampC* activator *ampR* (PSPA7_0985) and negative regulators *ampD* (PSPA7_5139) as well as putative regulators *ampDh3* (PSPA7_4711) and *ampDh2* (PSPA7_6284) are also present, as in PAO1 and PA14. Recently Dötsch et al. [18] screened a transposon mutant library of PA14 for mutants with increased resistance or susceptibility. They showed that mutants in PA14_37550 (PA2085) have 16-fold increased resistance to piperacillin and piperacillin-tazobactam. PA2085 is ubiquitous in sequenced strains except for PA7, where it is missing.

Fluoroquinolone (FQ) resistance is attributable to two point mutations, one in *gyrA* (Thr83Ile) and one in *parC* (Ser87Leu), known to be associated with FQ resistance [19]. There were several other differences with the corresponding PAO1 genes, and these differences are shared between PA7 and the PA7-related but fluoroquinolone-sensitive strain DSM1128. However, DSM1128 lacks the two FQ-related mutations.

The source of amikacin resistance is probably one of the efflux systems (see below). Although there is an *aacA4* gene (not present in PAO1 nor PA14) in an integron cassette inserted into a secondary site [16], the product is an AAC(6′)-II due to the substitution of a serine for a leucine at position 117 in PSPA7_3724.1 [20]. Several other aminoglycoside resistance determinants are present. Among these are the three PA7-specific genes in island RGP75 that constitute the center of Tn*5* and encode streptomycin, bleomycin and kanamycin resistance (PSPA7_3719-3720-3721). However, these three genes are not flanked by copies of IS*50*, which does not occur in the genome. Either these genes were mobilized (from PA7 or elsewhere) by tandem insertions of IS*50* to form Tn*5* or were left behind by precise excision of the IS50s. A second *aph (3′)-II* kanamycin resistance gene (PSPA7_0973) is present, as are two more streptomycin phosphotransferase genes, one a PA7-specific *aph (6)-Id* (PSPA7_5338) and the other an *str* gene (PSPA7_3432). PSPA7_2339 is an *arr* gene encoding an aminoglycoside response regulator. The *arr* gene is present in PAO1 but absent in PA14 and LESB58.

Chloramphenicol resistance is primarily due to the Mex efflux systems as in PAO1. The *catB7* gene PSPA7_4802 coding for a xenobiotic acetyltransferase of the hexapeptide-repeat superfamily similar to that found in other *P. aeruginosa* strains, a *catA* gene PSPA7_4187, and the major facilitator superfamily (MFS) exporter *cmx* gene PSPA7_3737.1 (the latter two PA7-specific) may also contribute to resistance. The *arnBCAD* operon (PSPA7_1593-1592-1591-1590) for resistance to polymyxin B and cationic antimicrobial peptides is present, as in PAO1 and PA14.

The major efflux pump MexABOprM and the secondary pump MexCDOprJ are intact in PA7, as are MexEFOprN and its regulator MexT, MexHIOpmD, MexMN, and MexVW. A “hybrid” system, MexXYOprA (PSPA7_3269-3270-3271) is present; the PA7-specific *oprA* gene is linked to *mexXY* rather than to *amrAB* as in *Burkholderia pseudomallei* [21]. Regulatory genes *mexR* (PSPA7_2746) and *mexZ* (PSPA7_3268) [22] are intact. An *oprD* gene is intact and presumably functional; PA7 is imipenem sensitive whereas *oprD* mutants are imipenem resistant [23]. Triclosan resistance is mediated by the general RND efflux systems, and the specific triclosan efflux system TriABC-OpmH [24] mediated by PSPA7_0234-0235-0236-5705 is also present. An MFS system, EmrAB-OpmG, is encoded by PSPA7_5897-5898-5896. A second putative system is coded by PSPA7_2115-2114-2113. Finally, PSPA7_5725 encodes an SMR multidrug efflux transporter.

### Virulence Factors

Perhaps the most striking feature of the whole PA7 genome is the lack of the entire 36-gene cluster corresponding to PA1690-PA1725 and encoding the type III secretion system. The *exoS*, *exoT*, and *exoY* genes encoding the “TTSS translocated effectors” are also deleted from the PA7 genome. In some strains, e.g. PA14, *exoU* substitutes for *exoS* [25], [26], but PA7 has neither; this is unusual among *P. aeruginosa* strains. A probable type II secretion system (PA2672-2677) is absent in PA7, while a novel one (PSPA7_1407-1420) is present in island RGP69. Also surprising is the deletion of the major exotoxin gene *toxA*. Additionally, genes encoding pyocins S2 and S5 are absent, and PA7 lacks the *plcD* gene encoding phospholipase D and the *rhlC* gene for rhamnolipid biosynthesis. However, *rhlA* (PSPA7_1647) and *rhlB* (PSPA7_1648) genes are present.

PA7 is similar to PAO1 and PA14 for most other factors identified in the Virulence Factor Database [27]. The 24 genes for synthesis and regulation of alginate are present, as is the 21-gene cluster (PSPA7_0142-0163; there is no 0153) for the Hcp secretion island-1 encoded type VI secretion system (H-T6SS). Genes for type IV pili and type IV twitching motility are similar to those of PAO1 and PA14.

The phenazine biosynthesis and modification genes are present except for *phzH*, which encodes a modifying enzyme that converts phenazine-1-carboxylic acid to phenazine-1-carboxamide. In one of the two copies of the *phzA-G* locus, *phzA1* and *phzB1* are fused (PSPA7_0888). The pyochelin genes are similar to those of the other sequenced strains, however, there are differences in the pyoverdin locus; PA7 lacks 4 genes relative to PAO1 but has in their place 3 genes (PSPA7_2826, 2859 and 2860) which are very similar to genes of C3719 and PACS-2. The putative *fpvA* pyoverdin receptor gene PSPA7_2861 is very similar to genes from strains 1-60 and 2-164; PA7 has no *fpvB* gene. Taken together, these results indicate that PA7 produces type II pyoverdin and has a type IIb *fpvA* gene [28], [29].

Virulence factors encoded by 1–3 genes have a one-to-one correspondence with PAO1 and PA14: alkaline protease (PSPA7_4143), elastase (PSPA7_1397 and PSPA7_3417), the GacS/GacA two-component system (PSPA7_4587 and PSPA7_2613), hemolytic phospholipase C *plcH* (PSPA7_4676), hydrogen cyanide production (PSPA7_3101-3103), non-hemolytic phospholipase C *plcN* (PSPA7_1801), phospholipase C *plcB* (PSPA7_0027), and protease IV (PSPA7_0919).

There are no *cupA* fimbrial genes in PA7; however, there are *cupB* and *cupC* genes as well as *cupD* genes. The latter are in genomic island RGP23, whereas those of PA14 are in its version of the PAPI-1 island [30]. As mentioned above, PA7 has an unusual glycosylation of its type IV pilin, mediated by TpfW [7], [8].

A study of signature-tagged mutagenesis mutants of LESB58 was performed by Winstanley et al. [4]. Of the 39 genes common to LESB58 and PAO1 identified as essential for lung infection, all are present except PA1721 (part of the TTSS cluster) and PA0325 (putative permease of ABC transporter).

### Quorum Sensing

The major genes of the quorum sensing system, *vqsM* (PSPA7_0474.1) [31] and *vqsR* (PSPA7_2608) [32] are present, as are the genes *lasR* (PSPA7_3898), *lasI* (PSPA7_3897), *rhlR* (PSPA7_1649), and *rhlI* (PSPA7_1650). One of the genes identified as a pseudogene because of an internal frameshift, PSPA7_4396, was *mvfR*, an important regulator of quorum sensing [33]. Éric Déziel (personal communication) confirmed the *mvfR* phenotype of PA7, and complemented the mutation by adding a plasmid containing the wild-type *mvfR* gene from PAO1. We believe that the lack of MvfR may be responsible for several of the differences seen in the preliminary proteomics experiment below.

### Proteomics

**Figure 3:**
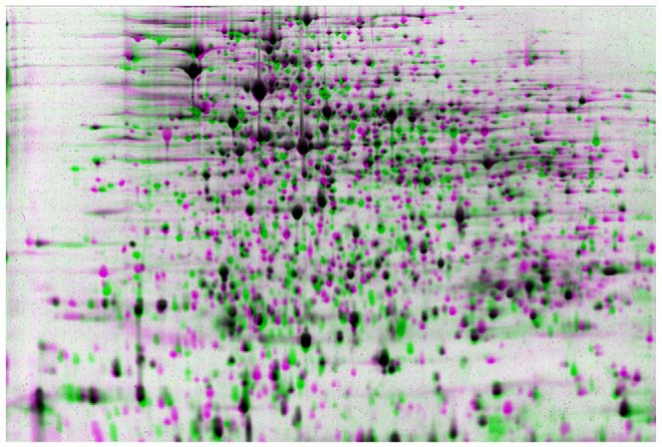
Proteomic comparison of PA7 and PAO1, Condition-level matching using Progenesis PG240 software. Green, PAO1-specific; pink, PA7-specific, black, PAO1-PA7 match.

Preliminary proteomics experiments were carried out using 2-D gels on PA7 and PAO1 (Figure 3). Among the interesting results are a 9-fold overexpression of the MucD serine protease and a 7.4-fold overexpression of anthranilate synthase in PA7 relative to PAO1. The latter is due to the genes in the tryptophan biosynthetic pathway (PSPA7_0753 and PSPA7_0790 for components I and II, respectively), not those in the *Pseudomonas* quinolone signal (PQS) pathway (PSPA7_4397 and PSPA7_4395). This may be related to the lack of the regulator MvfR noted above. Other significant differences include a 4.7-fold down-regulation of the chemotaxis protein CheZ and a 3.1-fold down-regulation of the outer membrane protein OmpA.

## Discussion

The complete genomic sequence of *P. aeruginosa* PA7 reveals that it is indeed a taxonomic outlier, and limited sequence data from housekeeping genes indicate that a certain number of other strains form a clade with PA7. It was noted by Mathee et al. [3] that of 7 genes used for multilocus sequence typing (*acsB*, *aroE*, *guaA*, *mutL*, *nuoD*, *ppsA*, and *trpE*) only *guaA*, although 99% identical between PAO1 and PA14, had 11 differences, enough to be phylogenetically useful. PA7 has only 86%–94% nucleotide identity with five of these genes; only *guaA* and *nuoD* being highly (97%–99%) conserved (Figure S2 A–G). Although PA7 is almost at the limit of a species, its rRNA genes and other genes of the protein synthesis machinery place it clearly within the species *P. aeruginosa*. It is possible that restriction-modification systems encoded by the genomic islands contribute to the genetic isolation of the strain.

PA7 has 18 unique genomic islands (also called “regions of genomic plasticity” or RGPs), as compared to 1–5 for the other sequenced strains. Of the 52 previously described RGP's [3], PA7 has genes in 33 of the loci, while 19 are empty in PA7. Among the occupied loci are RGP26 and RGP38 (previously unique to PA14), RGP8 (previously unique to C3719) and RGP12 (previously unique to PA2192). The contents of some islands are translocated in PA7, notably the pKLC102-like island in RGP7.

PA7 is highly resistant to most antibiotics, with the notable exception of carbapenems. However, resistance to carbapenems is increasingly frequent in *P. aeruginosa*, and is usually mediated by a plasmid-encoded VIM-type Beta-lactamase. An example is a VIM-8 producing serotype O12 isolate from South America [34]. Most resistance genes, notably those encoding the RND efflux systems, the AmpC and PoxB Beta-lactamases, and fluoroquinolone resistance due to point mutations in topoisomerase genes, are encoded by the core genome. Some resistance genes are on genomic islands, such as the aminoglycoside resistance genes on the putative integrated plasmid in RGP75, mercury resistance in a putative transposon in RGP63, an efflux system in RGP70, and streptomycin and sulfonamide resistance in RGP42.

PA7 is remarkably deficient in some key virulence factors, notably the whole TTSS locus, as well as its translocated effectors. ToxA exotoxin and pyocins S2 and S5 are also absent. However, several other virulence factor genes are intact. Quorum sensing is disrupted by a frameshift mutation in the *mvfR* gene. However, even some CF strains are deficient in quorum sensing (by *lasR* rather than *mvfR* mutations), and PA7 does come from a human infection, although not a respiratory infection. Future proteomics experiments on PA7 can include an *mvfR*-complementing plasmid, to distinguish the effects of QS deficiency from other regulatory differences between PA7 and PAO1 and/or PA14.

PA7 is a member of serotype O12, and the O-antigen locus is very similar to the sequenced O12 locus [9]. An epidemic of *P. aeruginosa* serotype O12, possibly of South Asian origin, occurred in Europe [35]. One study indicated a connection between overexpression of the AmpC Beta-lactamase with reduced expression of cell-to-cell signaling dependent virulence factors [36]. Speculatively, this may be related to PA7 and its QS deficiency.

It would be interesting to determine a second genomic sequence of a member of the PA7 clade. We have obtained a CF strain that is a member of this clade, and comparison of its core genome, and especially of its genomic islands, would be useful in determining the genomic basis for differences in virulence.

## Methods

### Minimum Inhibitory Concentration (MIC) Determination

The minimal inhibitory concentration (MIC) determination method was used to test the susceptibility of *P. aeruginosa* PA7 to a range of antibiotics. A serial twofold dilution series was generated for each antibiotic using appropriate starting concentrations in 3 ml of Müller–Hinton broth (Difco). The MIC corresponded to the smallest antibiotic concentration preventing the growth of *P. aeruginosa* PA7.

### Genome Sequencing and Annotation

The complete genome sequence of *P. aeruginosa* strain PA7 was determined using the whole-genome shotgun method as previously described [37]. Physical and sequencing gaps were closed using a combination of primer walking, generation and sequencing of transposon-tagged libraries of large-insert clones, and multiplex PCR [38]. Identification of putative protein-encoding genes and annotation of the genome were performed as previously described [39]. An initial set of genes predicted to encode proteins was identified with GLIMMER [40]. Genes consisting of fewer than 30 codons and those containing overlaps were eliminated. Frame shifts and point mutations were corrected or designated ‘authentic’ based on manual examination of the sequence trace files. Functional assignment, identification of membrane-spanning domains, and determination of paralogous gene families were performed as previously described [37]–[39].

### Trinucleotide Composition

Distribution of all 64 trinucleotides (3-mers) was determined, and the 3-mer distribution in 1,000-bp windows that overlapped by half their length (500 bp) across the genome was computed [39]. For each window, we computed the χ^2^ statistic on the difference between its 3-mer content and that of the whole chromosome. A large value for χ^2^ indicates the 3-mer composition in this window is different from the rest of the chromosome (minimum of two standard deviations). The assumptions inherent in this analysis are that the DNA composition is relatively uniform throughout the genome, and that 3-mer composition is independent.

### Comparative Genomics

The *P. aeruginosa* PA7 genome was compared to other *P. aeruginosa* genomes at the nucleotide level by suffix tree analysis using MUMmer [41], and the predicted PA7 CDSs were compared with the gene sets from the other sequenced *P. aeruginosa* genomes by BLAST using an E value cutoff of 1×10^−5^ and by HMM paralogous family searches using appropriate cutoffs established for each specific HMM. Available *P. aeruginosa* genomes were also compared via alignment using MAUVE [42] to determine the pairwise percentage identity of syntenous regions between PA7 and PAO1, PA14, and LESB58.

### Proteomics

Overnight cultures of strains PA7 and PAO1 were diluted in LB (1%) and harvested at similar culture densities (semi-log phase, OD_600_ ∼0.5). Cells were washed two times with PBS, and pellets were resuspended in 700 µL of 2D lysis buffer (20 mM Tris-HCl, pH 7.5, 7 M urea, 2 M thiourea, 3% CHAPS, 20 mM DTT, 5 mM Tris-(2-carboxyethyl)phosphine (TCEP), 0.5% IPG buffer pH 4–7 (GE Healthcare), and 0.25% IPG buffer pH 3–10 (GE Healthcare)) and incubated at RT for 2 h (vortexed every 15 min). Samples were centrifuged at 13,000×g for 5 min to remove insoluble materials, and proteins were precipitated with the 2D Clean-up kit (GE Healthcare) to remove substances interfering with IEF. Proteins were quantitated using the 2D Quant Kit (GE Healthcare). Two dimensional gel electrophoresis was carried out. In the first dimension, 150 µg of protein samples were run on 24 cm Immobiline DryStrips (GE Healthcare) of pH range 4.0–7.0 on an IPGphorII IEF system (GE Healthcare) as recommended by the manufacturer. Strips were equilibrated in equilibration buffer (50 mM Tris-HCl, pH 8.8, 6 M urea, 30% glycerol, 2% SDS, and a trace of bromophenol blue) containing 10 mg/mL DTT for 15 min and then in equilibration buffer containing 25 mg/mL iodoacetamide for 15 min, and sealed to 12% acrylamide gels made in-house using 0.5% agarose in standard Tris-glycine electrophoresis buffer. Second dimension was performed using the Ettan™ DALTtwelve system (GE Healthcare) at 17 W/gel and 25°C until the tracking dye was run off the gel. Proteins were visualized by Sypro Ruby fluorescence (Invitrogen). Gels were fixed overnight in 40% methanol and 7% acetic acid, stained for a minimum of 5 h, and then destained in 10% methanol and 7% acetic acid for 3×1 h. Gels were imaged with the ProXpress CCD camera-based scanner (Perkin-Elmer) at 100 µm resolution using 480 nm excitation and 620 nm emission filters. For each strain, 2D gels of 4 independent samples were analyzed using Progenesis PG240 v. 2006 (Nonlinear Dynamics). Spots were quantified with this software using the INCA processing algorithm and automatically matched using default setting. Matches were confirmed, and matching normalized spot volumes were compared by *t* test within the software to generate *p*-values. Gel plugs containing the proteins of interest were excised using a ProXcision robot (Perkin-Elmer) and subjected to LC-MS/MS analyses (Eastern Quebec Proteomics Centre, Centre Hospitalier de l'Université Laval, Quebec). Gel plugs were placed in 96-well plates and then washed with water. Tryptic digestions were performed on a MassPrep liquid handling robot (Micromass) according to the manufacturer's specifications and using sequencing grade modified trypsin (Promega). After extraction from the gel into 50% acetonitrile/0.1% formic acid, peptides were lyophilized in a speed vacuum and resuspended in 10 µL of 0.1% formic acid solution. Peptide MS/MS spectra were obtained by capillary liquid chromatography (10 cm, 75 µm picofrit column) coupled with an LTQ (ThermoFinnigan, San Jose, CA) quadrupole ion trap mass spectrometer with a nanospray interface. Resulting MS/MS spectra were interpreted using MASCOT (Matrix Science, London, UK; version 2.2.0) and searched against eubacterial proteins in the UniFef100 database. Carbamidomethylation of cysteine and partial oxidation of methionine, 2 missed cleavages, and an error tolerance of 2.0 Da for peptides and 0.5 Da for fragments were considered in the search. Scaffold (Proteome Software Inc., Portland, OR, USA; version 2.01.01) was used to validate MS/MS-based peptide protein identification.

### GenBank Accession

The complete annotated genome sequence is available at GenBank accession number CP000744.

## Supporting Information

Figure S1 Mauve alignment of the four *Pseudomonas aeruginosa* genomes PA7, PAO1, PA14 and LESB58. The height of the column alignment entropy bars show the degree of variation between conserved genes in these strains.
(0.10 MB TIF)

Figure S2 Phylogenetic trees based on the MLST schema genes for (A) AcsA, (B) AroE, (C) GuaA, (D) MutL, (E) NuoD, (F) PpsA and (G) TrpE respectively. These Neighbor-Joining gene trees were generated from protein sequences aligned with ClustalX outgrouped with homologs from *Vibrio cholerae* N16961 except for NuoD, which uses the *Shewanella oneidensis* MR-1 homolog as there isn't one in *V. cholerae* N16961. The outer nodes are labeled with Uniprot codes. The cluster representing the *Pseudomonas aeruginosa* species is shown with a vertical bar, *P. aeruginosa* PA7 is underlined. PSEA7 is PA7, PSEAB is PA14, PSEA8 is LESB58, PSEAE is either PAO1, PA2192, or C3719 (very similar *P. aeruginosa* members). These genes are used for the MLST schema, and are thus housekeeping genes evenly distributed around the genome, and on that basis reflect the evolutionary history of the genomes. Numbers at the inner nodes are bootstrap values generated from 1000 iterations of the bootstrap procedure.
(0.09 MB PDF)
