# Supplementary figures and images for: Complete Genome Sequence of the Multiresistant Taxonomic Outlier Pseudomonas aeruginosa PA7
Source: PLoS One. 2010 Jan 22;5(1):e8842. doi: 10.1371/journal.pone.0008842 (PMC2809737; doi:10.1371/journal.pone.0008842)

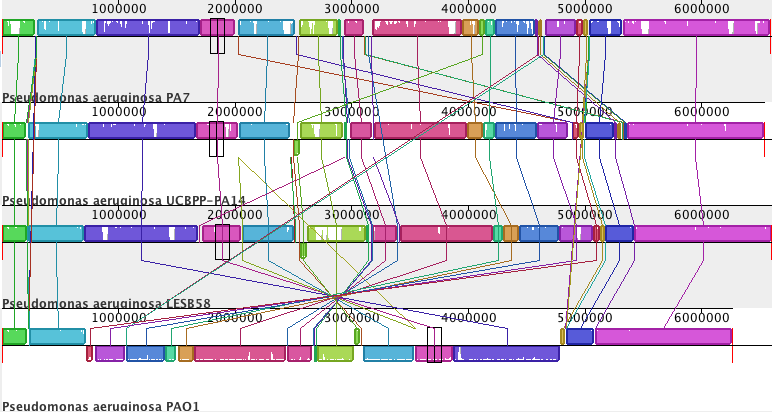

Supplement: Figure S1 — Mauve alignment of the four Pseudomonas aeruginosa genomes PA7, PAO1, PA14 and LESB58. The height of the column alignment entropy bars show the degree of variation between conserved genes in these strains. (0.10 MB TIF) [file pone.0008842.s001.tif]

FIGURE S2

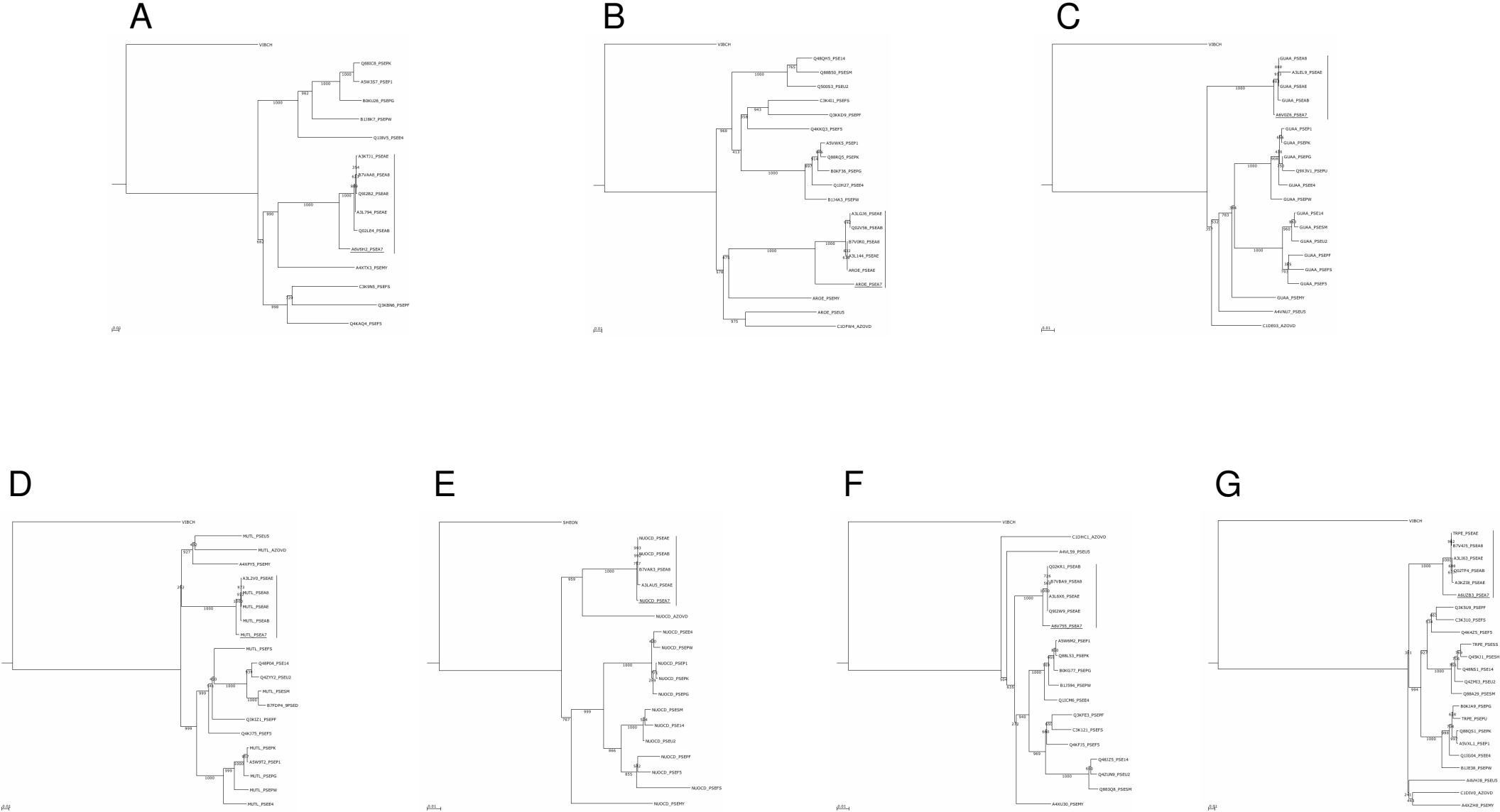

Supplement: Figure S2 — Phylogenetic trees based on the MLST schema genes for (A) AcsA, (B) AroE, (C) GuaA, (D) MutL, (E) NuoD, (F) PpsA and (G) TrpE respectively. These Neighbor-Joining gene trees were generated from protein sequences aligned with ClustalX outgrouped with homologs from Vibrio cholerae N16961 except for NuoD, which uses the Shewanella oneidensis MR-1 homolog as there isn't one in V. cholerae N16961. The outer nodes are labeled with Uniprot codes. The cluster representing the Pseudomonas aeruginosa species is shown with a vertical bar, P. aeruginosa PA7 is underlined. PSEA7 is PA7, PSEAB is PA14, PSEA8 is LESB58, PSEAE is either PAO1, PA2192, or C3719 (very similar P. aeruginosa members). These genes are used for the MLST schema, and are thus housekeeping genes evenly distributed around the genome, and on that basis reflect the evolutionary history of the genomes. Numbers at the inner nodes are bootstrap values generated from 1000 iterations of the bootstrap procedure. (0.09 MB PDF) [file pone.0008842.s002.pdf]
